# Supplementary material for: Protein structural disorder of the envelope V3 loop contributes to the switch in human immunodeficiency virus type 1 cell tropism
Source: PLoS One. 2017 Oct 19;12(10):e0185790. doi: 10.1371/journal.pone.0185790 (PMC5648111; doi:10.1371/journal.pone.0185790)
Supplement: S1 Fig — (*p<0.05, **p<0.01, ***p<0.001, asterisks colored with blue and red represent results for R5 and X4 virus, respectively; X means p>0.05). (PDF) [file pone.0185790.s003.pdf]

Supplementary figure

Fig S1. Boxplot and nonparametric comparisons of the V3 loop disorder tendency between R5 and X4 viruses from all patients (1, 2, 3, 5, 6, 7, 8, 9 and 11) in Shankarappa et al. data (1). (*p<0.05, **p<0.01, ***p<0.001, asterisks colored with blue and red represent results for R5 and X4 virus, respectively; X means p>0.05).

1. **Shankarappa R, Margolick JB, Gange SJ, Rodrigo AG, Upchurch D, Farzadegan H, Gupta P, Rinaldo CR, Learn GH, He X, Huang XL, Mullins JI.** 1999. Consistent viral evolutionary changes associated with the progression of human immunodeficiency virus type 1 infection. Journal of virology **73:**10489-10502.

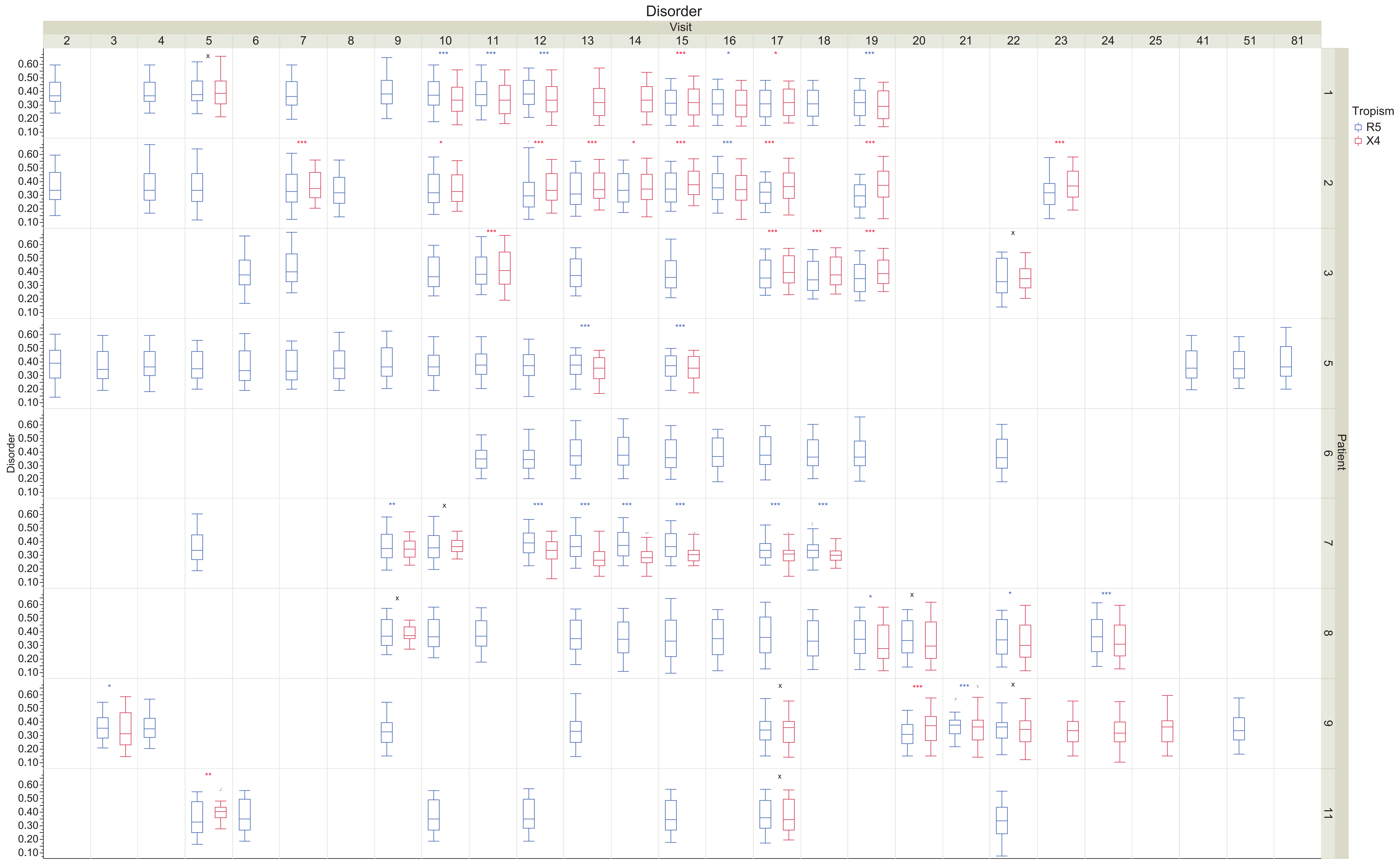

R5: * p<0.05 **p<0.01 ***p<0.001

X4: * p<0.05 **p<0.01 ***p<0.001
